# Identification of key genes and upstream regulators in ischemic stroke

**DOI:** 10.1002/brb3.1319

**Published:** 2019-06-06

**Authors:** Qian Zhang, Wenjie Chen, Siqia Chen, Shunxian Li, Duncan Wei, Wenzhen He

**Affiliations:** ^1^ Department of Pharmacy First Affiliated Hospital of Shantou University Medical College Shantou China; ^2^ Department of Neurology First Affiliated Hospital of Shantou University Medical College Shantou China

**Keywords:** differentially expressed genes, integrated analysis, ischemic stroke, transcription factors

## Abstract

**Introduction:**

Ischemic stroke (IS) causes severe neurological impairments and physical disabilities and has a high economic burden. Our study aims to identify the key genes and upstream regulators in IS by integrated microarray analysis.

**Methods:**

An integrated analysis of microarray studies of IS was performed to identify the differentially expressed genes (DEGs) in IS compared to normal control. Based on these DEGs, we performed the functional annotation and transcriptional regulatory network constructions. Quantitative real‐time polymerase chain reaction (qRT‐PCR) was performed to verify the expression of DEGs.

**Results:**

From two Gene Expression Omnibus datasets obtained, we obtained 1526 DEGs (534 up‐regulated and 992 down‐regulated genes) between IS and normal control. The results of functional annotation showed that Oxidative phosphorylation and Alzheimer's disease were significantly enriched pathways in IS. Top four transcription factors (TFs) with the most downstream genes including *PAX4*, *POU2F1*, *ELK1,* and *NKX2‐5*. The expression of six genes (*ID3*, *ICAM2*, *DCTPP1*, *ANTXR2*, *DUSP1,* and *RGS2*) was detected by qRT‐PCR. Except for *DUSP1* and *RGS2*, the other four genes in qRT‐PCR played the same pattern with that in our integrated analysis.

**Conclusions:**

The dysregulation of these six genes may involve with the process of ischemic stroke (IS). Four TFs (*PAX4*, *POU2F1*, *ELK1* and *NKX2‐5*) were concluded to play a role in IS. Our finding provided clues for exploring mechanism and developing novel diagnostic and therapeutic strategies for IS.

## 1 INTRODUCTION

Stroke is one of the leading causes of serious long‐term disability and mortality worldwide, which is characterized by symptoms such as inability to move or feel one side of the body, inability to speak, or understand problems and dizziness (Donnan, Fisher, Macleod, & Davis, 2008; Dorrance & Fink, 2015). Ischemic stroke (IS) is one of the main types of stroke, which is characterized by cerebral ischemia. Survival of IS can be lead to severe neurological impairments and physical disabilities with a high economic burden (Evers et al., 2004; Volny, Kasickova, Coufalova, Cimflova, & Novak, 2015). Up to now, thrombolysis is the only efficacious treatment for IS (Chi & Chan, 2017). Despite recent progress, little is known about the underlying pathophysiology mechanisms of IS and much work still needs to be done to fully elucidate the pathophysiology mechanisms.

With high‐throughput genetic analysis, the emergence of gene expression profiles has became an effective method to identify differentially expressed genes (DEGs) in a variety of diseases which help to explore pathogenesis and develop biomarkers(Alieva et al., 2014; Du, Yang, Tian, Wang, & He, 2014; Kong et al., 2018). Due to differences of samples and platforms in multiple microarray studies, integrated analysis of multiple microarray studies can identify more accurate profiles of DEGs with a larger sample size than a single microarray. Exploring the upstream transcription factors (TFs) mediating abnormal gene expression in disease status can help to understand pathophysiological changes in complex diseases (Li, Dani, & Le, 2009; Zhao, Wang, Xu, Li, & Yu, 2017).

Our study aims to make an integrated analysis of multiple IS microarray data to obtain the key DEGs in the pathogenesis of IS. Functional annotation and protein and IS‐specific transcriptional regulatory network construction were performed to explore the biological functions of DEGs, which hopefully provide clues for exploring mechanism and developing novel diagnostic and therapeutic strategies for IS.

## 2 MATERIALS AND METHODS

### 2.1 Microarray expression profiling in Gene Expression Omnibus

In this study, we searched datasets from the Gene Expression Omnibus (GEO) database (http://www.ncbi.nlm.nih.gov/geo/) with the keywords "ischemic stroke, IS"[MeSH Terms] OR "ischemic stroke, IS" [All Fields]) AND "Homo sapiens"[porgn] AND "gse"[Filter]. The inclusion criteria for this study were: (a) The type of dataset was described as “expression profiling by array. (b) Dataset should be whole‐genome mRNA expression profile by array. (c) Datasets were obtained by blood samples of IS and normal control group (no drug stimulation or transfection). (d) The datasets should be normalized or original, and two sets of mRNA data (GSE22255 and GSE16561) of IS were selected. In GSE22255, Gene expression profiling was performed in peripheral blood mononuclear cells of 20 IS patients and 20 sex‐ and age‐matched controls using Affymetrix microarrays. In GSE16561, total RNA extracted from whole blood in 39 IS patients compared to 24 healthy control subjects.

### 2.2 Identification of DEGs between IS and normal controls

MetaMA, an R package, is used to combine data from multiple microarray datasets, and we obtained the individual *p*‐values. The Benjamini & Hochberg method were used to obtain multiple comparison correction false discovery rate (FDR). DEGs in IS compared to normal controls were obtained with FDR < 0.05. The heat‐map of top 100 DEGs was obtained by pheatmap package.

### 2.3 Functional annotation of DEGs

By using GeneCodis3 (http://genecodis.cnb.csic.es/analysis), gene ontology (GO) enrichment analysis and Kyoto Encyclopedia of Genes and Genomes (KEGG) pathway enrichment analysis were performed to uncover biological functions of DEGs. FDR < 0.05 was considered as statistically significant.

### 2.4 Construction of IS‐specific transcriptional regulatory networks

Based on the integrated analysis, the corresponding promoters of the top 20 up‐regulated or down‐regulated DEGs were obtained by UCSC (http://genome.ucsc.edu). The TFs involved in regulating these DEGs were derived from the match tools in TRANSFAC. The IS‐specific transcriptional regulatory network was constructed by Cytoscape software (http://www.cytoscape.org/).

### 2.5 Validation the expression of DEGs in qRT‐PCR

Five adult patients with IS and five normal controls were enrolled in our study from First Affiliated Hospital of Shantou University Medical College. Patients with neurological diseases, cardiac embolism, transient ischemic attack, hemorrhagic infarction, occult cerebrovascular malformation, traumatic cerebrovascular disease were excluded. Normal control without history of stroke, head trauma and surgery, heart surgery or neurological disease was included in this study. All subjects were first on an empty stomach for 12 hr. Then, we collected the blood samples by venipuncture at 7:00–8:00 of the next morning. The patient demographics, clinical features and risk factors were extracted from medical records as displayed in Table 1. Informed written consent was obtained from all participants, and research protocols were approved by the ethical committee of First Affiliated Hospital of Shantou University Medical College. The criteria of choosing candidate genes as follow: In Top five up/down DEGs, we randomly selected six genes. Therefore, ID3, ICAM2, DCTPP1, ANTXR2, DUSP1, and RGS2 were selected as the validation DEGs.

**Table 1:** Subject characteristics

	Case‐1	Case‐2	Case‐3	Case‐4	Case‐5	Control‐1	Control‐2	Control‐3	Control‐4	Control‐5
Race	Asian	Asian	Asian	Asian	Asian	Asian	Asian	Asian	Asian	Asian
Gender/ Age	Female/68	Female/44	Male/64	Female/76	Female/77	Male/50	Male/61	Female/77	Male/72	Female/50
GLU (mmol/L)	12.11	4.74	4.63	8.44	6.8	4.97	4.47	5.98	6.03	5.19
TG (mmol/L)	2.35	0.85	2.04	1.87	4.32	1.94	0.42	1.99	1.53	1.08
HDL‐C (mmol/L)	0.67	0.95	1.09	0.92	1.98	1.4	1.29	0.94	0.89	1.57
LDL‐C (mmol/L)	2.45	1.74	1.91	3.05	1.01	3.35	2.53	2.98	2.94	3.82
Hypertension	Yes	No	Yes	Yes	Yes	No	No	No	No	No
Diabetes	Yes	Yes	No	Yes	Yes	Yes	No	No	No	No
Hyperlipidemia	Yes	No	Yes	Yes	No	Yes	No	No	No	No
Smoker	No	No	No	No	No	No	No	No	Yes	No
Drinking	No	No	No	No	No	No	No	No	Yes	No

Abbreviations: GLU, glucose; HDL‐C, high‐density lipoprotein cholesterol; LDL‐C, low‐density lipoprotein cholesterol; TG, triglyceride.

Total RNA was extracted with a RNA simple total RNA kit (Tiangen, China). RNA (2 μg) was reverse‐transcribed using a Fast Quant RT Kit (Tiangen, Beijing, China) according to the manufacturer's instructions. Quantitative real‐time PCR were conducted using the Super Real PreMix Plus SYBR Green (Tiangen, China) on ABI 7500 real‐time PCR system. The amplification process was performed under the following conditions: 15 min at 95°C followed by 40 cycles of 10 s at 95°C, 30 s at 55°C, 32 s at 72°C, and 15 s at 95°C, 60 s at 60°C, 15 s extension at 95°C. Relative quantification of mRNA levels was analyzed by using the 2−∆∆Ct method. Each sample was analyzed in triplicate. The human GAPDH were used as endogenous controls for gene expression in analysis. The PCR primers used are listed in Table 2.

**Table 2:** Primer sequences used for qRT‐PCR

Name	Sequence (5′ to 3′)
GAPDH‐F	GGAGCGAGATCCCTCCAAAAT
GAPDH‐R	GGCTGTTGTCATACTTCTCATGG
ID3‐F	GAGAGGCACTCAGCTTAGCC
ID3‐R	TCCTTTTGTCGTTGGAGATGAC
ICAM2‐F	CGGATGAGAAGGTATTCGAGGT
ICAM2‐R	CACCCACTTCAGGCTGGTTAC
DCTPP1‐F	CGCCTCCATGCTGAGTTTG
DCTPP1‐R	CCAGGTTCCCCATCGGTTTTC
ANTXR2‐F	GATCTCTACTTCGTCCTGGACA
ANTXR2‐R	AAATCTCTCCGCAAGTTGCTG
DUSP1‐F	ACCACCACCGTGTTCAACTTC
DUSP1‐R	TGGGAGAGGTCGTAATGGGG
RGS2‐F	AAGATTGGAAGACCCGTTTGAG
RGS2‐R	GCAAGACCATATTTGCTGGCT

Abbreviation: qRT‐PCR, quantitative real‐time polymerase chain reaction.

## 3 RESULTS

### 3.1 DEGs in IS

**Figure 1:**
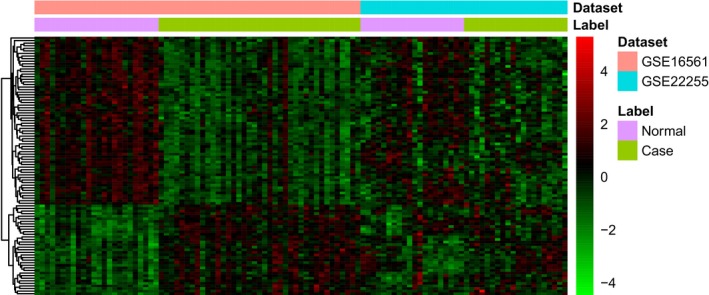
The heat‐map of top 100 DEGs in IS compared to normal control. Row and column represented DEGS and GEO data, respectively. The color scale represented the expression levels. DEGs, differentially expressed genes; GEO, Gene Expression Omnibus; IS, ischemic stroke

Two datasets (GSE22255 and GSE16561) were downloaded from GEO (Table 3). Samples of GSE22255 and GSE16561 were obtained from participants of Portugal and USA, respectively. Compared with the normal controls, 1526 DEGs in IS were obtained with FDR < 0.05, among which, 534 genes were up‐regulated and 992 genes were down‐regulated. Top 20 DEGs between IS and normal controls were displayed in Table 4. Heat map of top 100 DEGs was displayed in Figure 1.

**Table 3:** Gene expression datasets used in this study

Type	GEO ID	Platform	Sample count (N:P)	Notes	(Refs.)
mRNA	GSE22255	GPL570[HG‐U133_Plus_2] Affymetrix Human Genome U133 Plus 2.0 Array	40 (20:20)	Oliveira SA, Portugal, 2011	(Krug et al., 2012)
mRNA	GSE16561	GPL6883 Illumina HumanRef‐8 v3.0 expression beadchip	63 (24:39)	Barr TL, USA, 2010	(Barr et al., 2010)

Abbreviation: GEO, Gene Expression Omnibus.

**Table 4:** The top 20 DEGs in IS

ID	Symbol	Combined.ES	P.Value	FDR	Regulation
3399	*ID3*	−1.41974	8.42E‐10	4.33E‐06	Down
3384	*ICAM2*	−1.32659	2.18E‐09	4.33E‐06	Down
79077	*DCTPP1*	−1.36213	2.58E‐09	4.33E‐06	Down
8270	*LAGE3*	−1.31394	4.31E‐09	6.02E‐06	Down
1677	*DFFB*	−1.32119	1.14E‐08	1.19E‐05	Down
7693	*ZNF134*	−1.26287	2.46E‐08	2.05E‐05	Down
10838	*ZNF275*	−1.22866	2.57E‐08	2.05E‐05	Down
10450	*PPIE*	−1.22151	2.89E‐08	2.11E‐05	Down
353	*APRT*	−1.25353	4.45E‐08	2.96E‐05	Down
128240	*APOA1BP*	−1.23072	4.69E‐08	2.96E‐05	Down
118429	*ANTXR2*	1.573782	7.94E‐10	4.33E‐06	Up
1843	*DUSP1*	1.313089	1.71E‐09	4.33E‐06	Up
84898	*PLXDC2*	1.514479	1.82E‐09	4.33E‐06	Up
5997	*RGS2*	1.518917	1.89E‐09	4.33E‐06	Up
728	*C5AR1*	1.420349	2.23E‐09	4.33E‐06	Up
4082	*MARCKS*	1.423355	2.42E‐09	4.33E‐06	Up
25909	*AHCTF1*	1.349131	2.55E‐09	4.33E‐06	Up
136319	*MTPN*	1.420982	4.21E‐09	6.02E‐06	Up
8349	*HIST2H2BE*	1.307212	8.94E‐‐09	1.07E‐05	Up
329	*BIRC2*	1.287374	1.12E‐08	1.19E‐05	Up

Abbreviation: DEGs, differentially expressed genes.

### 3.2 Functional annotation of DEGs

**Figure 2:**
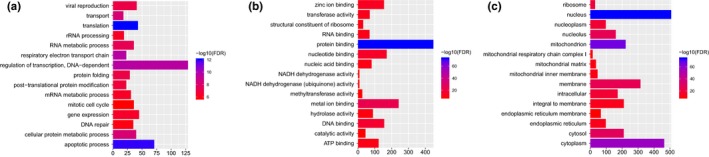
The top 15 most significantly enriched GO terms of DEGs in IS compared to normal control. The x‐axis shows −log FDR and y‐axis shows GO terms (a) Biological process. (b) Molecular function. (c) Cellular component. DEGs, differentially expressed genes; FDR, false discovery rate; GO, gene ontology; IS, ischemic stroke

**Figure 3:**
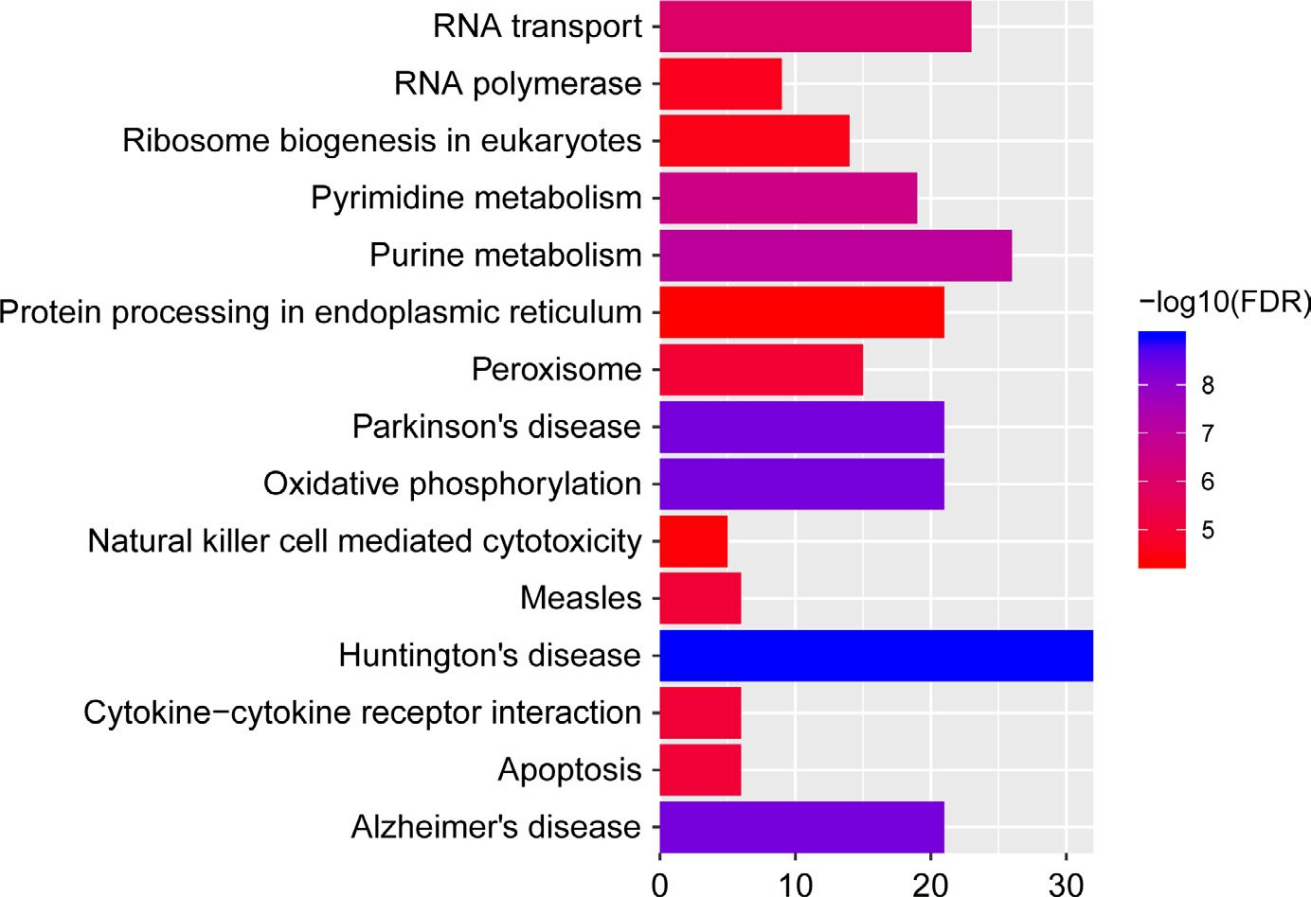
Top 15 significantly enriched pathways of DEGs in IS compared to normal control. The x‐axis shows −log FDR and y‐axis shows KEGG pathways. DEGs, differentially expressed genes; FDR, false discovery rate; IS, ischemic stroke; KEGG, Kyoto Encyclopedia of Genes and Genomes

According to the GO enrichment analysis with FDR < 0.05, apoptotic process (FDR = 1.25E‐12), respiratory electron transport chain (FDR = 2.58E‐10) and mitochondrion (FDR = 1.28E‐66) were significantly enriched GO terms. The top 15 GO terms of DEGs in IS were displayed in Figure 2. After the KEGG pathway enrichment analysis (FDR < 0.05), we found that Oxidative phosphorylation (FDR = 4.56E‐09) and Alzheimer's disease (AD) (FDR = 4.56E‐09) were significantly enriched pathways in IS. Top 15 most significantly enriched KEGG pathways of DEGs in IS were shown in Figure 3.

### 3.3 Construction of IS‐specific transcriptional regulatory networks

**Figure 4:**
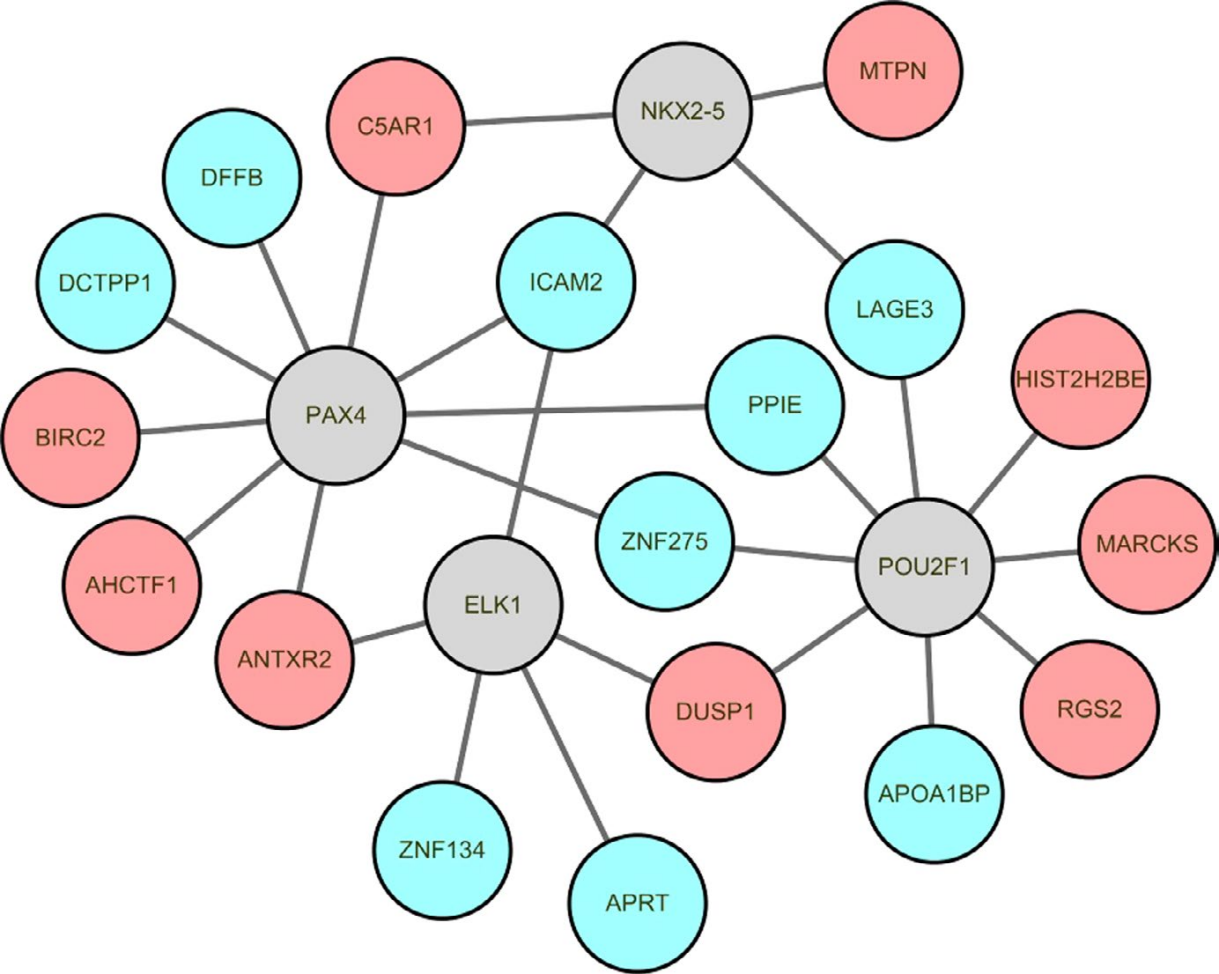
The IS‐specific transcriptional regulatory network. Red‐ and green‐color nodes represent the up‐ and down‐regulated DEGs targeted by TFs, respectively. Gray nodes denote the TFs which predicted to interact with the corresponding DEGs. DEGs, differentially expressed genes; IS, ischemic stroke; TFs, transcription factors

According to TRANSFAC, 35 TFs targeting 20 DEGs (top 10 up‐regulated or down‐regulated genes) were identified. Top four TFs with the most downstream genes (top 20 DEGs) including *PAX4*, *POU2F1*, *ELK1,* and *NKX2‐5* (Table 5). Top four TFs and their target gene regulatory network maps were built, which consisted of 22 nodes and 26 edges (Figure 4).

**Table 5:** Top 4 TFs that covered the most target genes

Transcription factor	ID	Count	Genes
*PAX4*	5078	9	*PPIE, ZNF275, ANTXR2, DFFB, AHCTF1, BIRC2, ICAM2, C5AR1, DCTPP1*
*POU2F1*	5451	8	*PPIE, ZNF275, APOA1BP, DUSP1, MARCKS, RGS2, LAGE3, HIST2H2BE*
*ELK1*	2002	5	*ANTXR2, DUSP1, ICAM2, APRT, ZNF134*
*NKX2‐5*	1482	4	*MTPN, ICAM2, C5AR1, LAGE3*

Abbreviation: TFs, transcription factors.

### 3.4 Validation the expression of DEGs in qRT‐PCR

**Figure 5:**
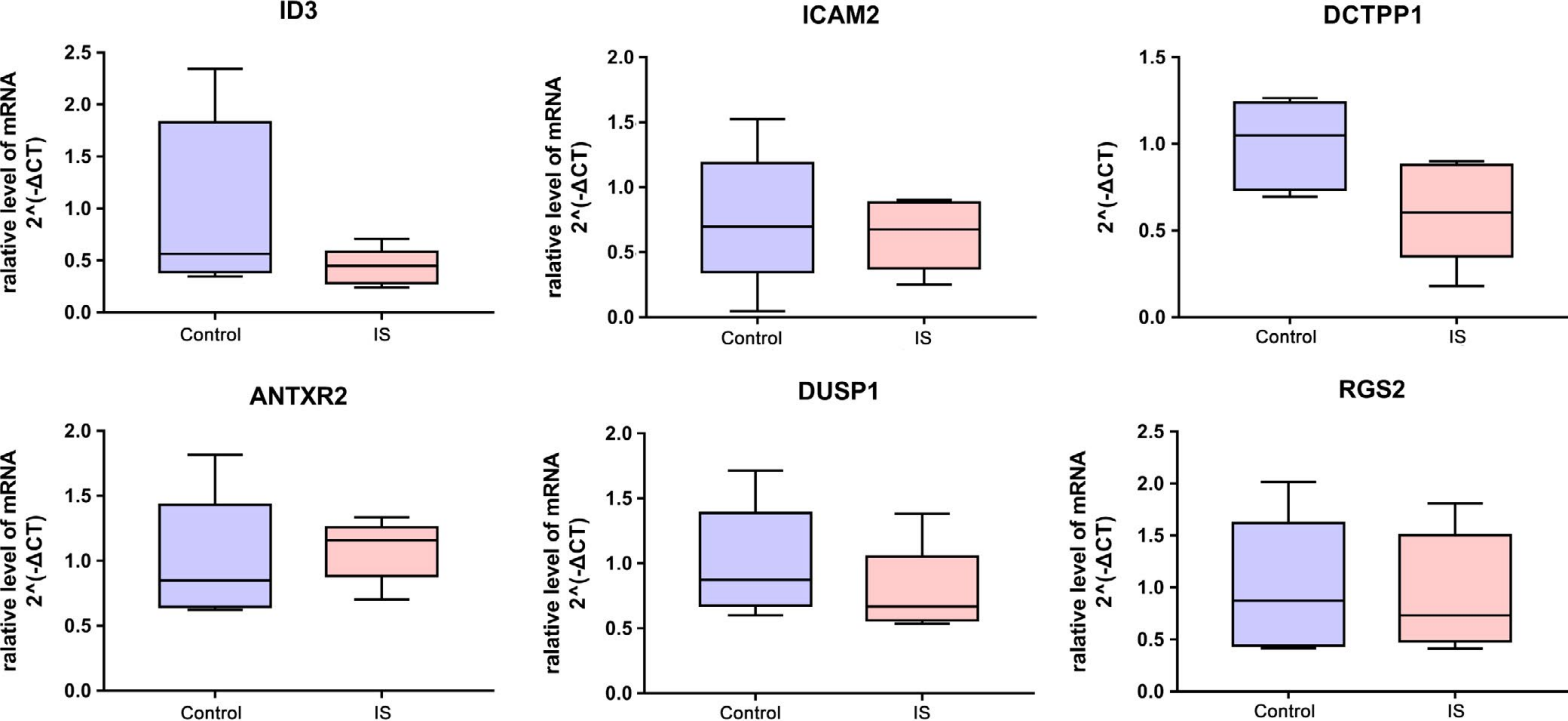
The validation of the expression levels of selected DEGs in IS. The x‐axis shows DEGs and y‐axis shows log2 (fold change) between IS and normal controls. DEGs, differentially expressed genes; IS, ischemic stroke

According to our integrated microarray analysis based on GEO, six DEGs including *ID3*, *ICAM2*, *DCTPP1*, *ANTXR2*, *DUSP1,* and *RGS2* were selected to perform the quantitative real‐time polymerase chain reaction (qRT‐PCR) confirmation (Figure 5). *ID3*, *ICAM2*, *DCTPP1*, *ANTXR2*, *DUSP1,* and *RGS2* were top 5 DEGs. Compared to normal control, *ID3*, *ICAM2* and *DCTPP1* were down‐regulated while ANTXR2 was up‐regulated in IS in the qRT‐PCR confirmation which was consistent with that in integrated microarray analysis. Compared to normal control, *DUSP1* and *RGS2* were down‐regulated in IS in qRT‐PCR confirmation while up‐regulated in IS in integrated microarray analysis results. We hypothesized that this inconsistence might be influenced by the small sample size of qRT‐PCR.

## 4 DISCUSSION

To better uncover the pathogenesis and develop novel diagnostic and therapeutic strategies for IS, we performed this integrated analysis between IS patients and normal controls. A total of 1526 genes across the studies were consistently differentially expressed in IS (534 up‐regulated and 992 down‐regulated) with FDR < 0.05. *PAX4*, *POU2F1*, *ELK1,* and *NKX2‐5* were top four TFs with the most downstream genes. *ID3*, *ICAM2*, *DCTPP1*, *ANTXR2*, *DUSP1,* and *RGS2*, which were top 10 up‐regulated or down‐regulated DEGs, were selected candidate gene to verify their expression in IS. Except for *DUSP1* and *RGS2*, the other four genes in qRT‐PCR played the same pattern with that in our integrated analysis, suggesting the results of our integration analysis are reliable.

As one of Inhibitor of DNA binding family, *ID3* have been known to regulate cell growth, self‐renewal, senescence, angiogenesis, and neurogenesis (Doke, Avecilla, & Felty, 2018). The *ID3* is biologically relevant to neurological and behavior research because of its involvement in the stress response, neural plasticity, and neural circuitry (Avecilla, Doke, & Felty, 2017). *ID3* was down‐regulated in peripheral blood of IS patient, and identified candidate gene that can accurately detect IS (O'Connell et al., 2016). The diagnostic robustness of the identified 10 candidate genes (*ID3* is one of 10 candidate genes) in an independent patient population, and further suggest that it is temporally stable over the first 24 hr of stroke pathology (O'Connell, Chantler, & Barr, 2017). In this study, the expression of *ID3* was down‐regulated in the blood of patients with IS in the results of bioinformatics analysis and qRT‐PCR validation. Our results provide further evidence that *ID3* may be an important biomarker for the diagnosis of IS.

Intercellular adhesion molecule‐2 (*ICAM‐2*) belongs to the *ICAM* family of adhesion proteins. *ICAM2* is expressed in vascular endothelial cells and blood cells, and plays a key role in cell‐cell interactions during humoral immunity (Lyck & Enzmann, 2015). *ICAM2* also promotes neutrophil binding to and migration through vascular endothelium as a component of immune reactions (Huang et al., 2006). Platelet‐leukocyte aggregation and platelet activation are found to be on the higher side in IS patients, and *ICAM2* is an important gene in regulating interaction of platelets with leukocytes. Herein, *ICAM2* was down‐regulated in the blood of patients with IS in the results of bioinformatics analysis and qRT‐PCR validation. Transcriptional regulatory networks results showed that *ICAM2* was co‐expression with *PAX4*, *ELK1* and *NKX2‐5*. Therefore, we speculated that *ICAM2* may be involved in the occurrence of IS.

ANTXR cell adhesion molecule 2 (*ANTXR2*), also known as *CMG2*, encodes a 55‐kDa type I transmembrane protein serves as capillary morphogenesis protein 2 (Youssefian et al., 2017). *ANTXR2* also known as the main receptor of the anthrax toxin (Scobie, Rainey, Bradley, & Young, 2003). *ANTXR2* was up‐regulated in peripheral blood of IS patient, and identified candidate gene that can accurately detect IS (O'Connell et al., 2016). In this study, the results of bioinformatics analysis and qRT‐PCR validation showed that *ANTXR2* was up‐regulated in IS patient. *ANTXR2* was co‐expression with *PAX4* and *ELK1* in transcriptional regulatory networks. These finds indicated *ANTXR2* may play a pivotal role in IS.

Base on functional annotation, apoptotic process, respiratory electron transport chain and mitochondrion were significantly enriched GO terms, and Oxidative phosphorylation and AD were significantly enriched pathways in IS. In mitochondrial genome‐wide association study of IS, a genetic score comprised of the sum of all common variants in the mitochondrial genome showed association with IS (Anderson et al., 2011). The associations for small vessel stroke and deep intracerebral hemorrhage suggest that genetic variation in oxidative phosphorylation influences small vessel pathobiology (Anderson et al., 2013). Oxidative phosphorylation plays an important role in neuronal response to oxidative damage (Nicholls, 2008). Is and AD, despite being distinct disease entities, share numerous pathophysiological mechanisms such as those mediated by inflammation, immune exhaustion, and neurovascular unit compromise (Lucke‐Wold et al., 2015). Therefore, we speculated that Oxidative phosphorylation and AD pathway may play an important role in pathogenesis of IS.

In conclusion, our study identified several DEGs, TFs, and pathways in IS which provides clues to understand the pathology and develop diagnostic and therapeutic targets for the IS. The new DEGs, TFs, and pathways of IS obtained in our integrated analysis suggested that integrated microarray analysis is a good way to uncover the molecular mechanism of diseases. However, there are limitations in this study. The number of samples for qRT‐PCR confirmation was small. Further research with larger sample size was performed to confirm our finding and explore the precise role of key DEGs in IS.

## CONFLICT OF INTEREST

None.

## DATA AVAILABILITY STATEMENT

The dataset supporting the conclusions of this article is included within the article.
